# A Vaccine Encoding Conserved Promiscuous HIV CD4 Epitopes Induces Broad T Cell Responses in Mice Transgenic to Multiple Common HLA Class II Molecules

**DOI:** 10.1371/journal.pone.0011072

**Published:** 2010-06-11

**Authors:** Susan Pereira Ribeiro, Daniela Santoro Rosa, Simone Gonçalves Fonseca, Eliane Conti Mairena, Edilberto Postól, Sergio Costa Oliveira, Luiza Guilherme, Jorge Kalil, Edecio Cunha-Neto

**Affiliations:** 1 Laboratory of Clinical Immunology and Allergy-LIM60, Division of Clinical Immunology and Allergy, Department of Medicine, University of São Paulo School of Medicine, São Paulo, Brazil; 2 Heart Institute (InCor), University of São Paulo School of Medicine, São Paulo, Brazil; 3 Institute for Investigation in Immunology-INCT, São Paulo, Brazil; 4 Department of Biochemistry and Immunology, Federal University of Minas Gerais, Belo Horizonte, Brazil; University of Toronto, Canada

## Abstract

Current HIV vaccine approaches are focused on immunogens encoding whole HIV antigenic proteins that mainly elicit cytotoxic CD8+ responses. Mounting evidence points toward a critical role for CD4+ T cells in the control of immunodeficiency virus replication, probably due to cognate help. Vaccine-induced CD4+ T cell responses might, therefore, have a protective effect in HIV replication. In addition, successful vaccines may have to elicit responses to multiple epitopes in a high proportion of vaccinees, to match the highly variable circulating strains of HIV. Using rational vaccine design, we developed a DNA vaccine encoding 18 algorithm-selected conserved, “promiscuous” (multiple HLA-DR-binding) B-subtype HIV CD4 epitopes - previously found to be frequently recognized by HIV-infected patients. We assessed the ability of the vaccine to induce broad T cell responses in the context of multiple HLA class II molecules using different strains of HLA class II- transgenic mice (-DR2, -DR4, -DQ6 and -DQ8). Mice displayed CD4+ and CD8+ T cell responses of significant breadth and magnitude, and 16 out of the 18 encoded epitopes were recognized. By virtue of inducing broad responses against conserved CD4+ T cell epitopes that can be recognized in the context of widely diverse, common HLA class II alleles, this vaccine concept may cope both with HIV genetic variability and increased population coverage. The vaccine may thus be a source of cognate help for HIV-specific CD8+ T cells elicited by conventional immunogens, in a wide proportion of vaccinees.

## Introduction

Given the proportions of the AIDS pandemic, development of an effective vaccine against human immunodeficiency virus type 1 (HIV-1) remains one of the most important biomedical research priorities. Only three vaccine concepts have completed clinical efficacy studies so far, with two negative results (Env gp 120 vaccine -AIDSVAX; recombinant Adenovirus 5 HIV-1 gag/pol/nef trivalent vaccine -STEP trial [1]), and one showing borderline efficacy, with no effect on HIV-1 viral load (recombinant canarypox ALVAC (gp120/Gag/Protease) prime - gp120 protein boost - ALVAC-AIDSVAX) [2].

Vaccination regimens that induced cell-mediated immunity were shown to significantly reduce simian immunodeficiency virus (SIV) replication in non-human primates [3], [4], [5]. Therefore, most of the HIV-1 vaccine field is currently focused on development of vaccines that elicit potent cytotoxic CD8+ responses. The lack of efficacy of the recently studied STEP trial of the Adenovirus 5 HIV-1 gag/pol/nef trivalent vaccine [6] may have been related to the narrowness of the induced T cell response – on average, only one epitope was recognized per HIV-1 gene product in each vaccinee [7]. Recent evidence from the SIV infected macaque model show that heterozygote animals, which carry more MHC molecules and present a broader T cell response, display better control of viral load than their homozygote counterparts [8]. It has thus been argued that the development of novel vaccine strategies that elicit a greater epitope breadth, matching T cell responses to circulating HIV strains, is a critical step to improve effectiveness of a vaccine against the highly variable HIV-1 [7]. In addition, 30% and 60% of vaccinees in the STEP trial failed to display CD8+ and CD4+ T cell responses to HIV epitopes, respectively [9]. Regarding the ALVAC-AIDSVAX trial, no CD8+ T cell responses were detected among the vaccinees, and 66% of them failed to display CD4+ T cell responses to gp120 [2]. Thus, vaccines tested in recent efficacy trials failed to induce CD8+ and especially CD4+ T cell responses in a significant proportion of vaccinees. The lack of population coverage may thus have been another cause of the insufficient results of the trials. Thus, innovative vaccine antigen design and immunogen formulation is needed in order to develop a vaccine able to induce broad immune responses in the majority of vaccinees. One possible way to increase the breadth of the response would be to include most or all of the HIV-1 proteome into a viral vector [5]. However, most viral vectors pose constraints on insert size, and developing such a vaccine for large-scale use could be technically challenging or impractical. On the other hand, epitope-based vaccines combine multiple T cell epitopes assembled in tandem, and can focus the immune response on any chosen group of epitopes (e. g. conserved and highly antigenic), generating much smaller insert sizes. Every single epitope can be immunogenic in multiple epitope-based vaccines; in addition, they have been reported to generate broad and potent immune responses [10], [11], [12], [13], [14]. By eliminating epitope flanking regions, epitope vaccines are also devoid of mutations that impair antigen processing and presentation, which may have accumulated in the flanking regions along viral evolution [15].

In spite of the abundant evidence that cytotoxic CD8+ T lymphocytes are the primary anti-HIV-1 effectors [16], several studies have shown that a strong specific CD4+ T-cell response is associated with control of viral replication and long-term nonprogression to AIDS [17], [18], [19], [20]. It has been shown that specific CD4+ T cells play a major role in the generation of a CD8+ cytotoxic T cell response and neutralizing antibodies, able to control viral replication [21], [22], [23]. Furthermore, CD4+ T cells contribute to T cell vaccine-induced protection in SIV-infected primates [24], [25]. Protective mechanisms include cognate help for functional CD8+ T cell memory [26], [27], mobilization of effector CD8+ T cells to peripheral sites of infection [28], and inhibition of SIV replication in infected macrophages [29]. In addition, virus-specific CD4+ T cells may be able to tolerate more sequence diversity in their target epitopes than CD8+ T cells, therefore being more resistant to mutational escape [30]. It follows that deliberate inclusion of HIV-1 CD4+ T cell epitopes in HIV-1 immunogens, to provide cognate help and boost CD8+ T cell responses, might be a desirable approach for prophylactic T cell vaccines.

With the aid of the TEPITOPE algorithm [31], our group has previously identified a set of 18 conserved CD4+ T-cell epitopes, derived from HIV-1 subtype B consensus whole proteome, capable of binding to multiple HLA-DR molecules [32]. The 18 peptides were recognized, and PBMC (peripheral blood mononuclear cells) from over 90% of HIV-1-infected patients displayed IFN-γ ELISPOT responses to the peptides; each patient recognized on average 5 peptides, including both CD4+ and CD8+ T cell responses [32]. A vaccine encoding multiple conserved epitopes may increase crossreactivity between different HIV strains, possibly circumventing HIV-1 genetic variability [1]. Furthermore, one would expect that a vaccine built with multiple “promiscuous” peptides, capable to bind to several HLA class II molecules could lead to an increased coverage of the genetically heterozygous human population. Since essentially all HLA class II molecules tested were shown to bind to multiple promiscuous HIV-1 epitopes [32], it is expected that most individuals could develop broad T cell responses. We thus hypothesized that an immunogen containing such a set of 18 conserved, highly promiscuous, immunodominant epitopes from 8 different HIV-1 proteins could potentially elicit a broad T cell response in a high proportion of individuals bearing distinct HLA class II molecules. In order to test our hypothesis, we designed a DNA vaccine encoding the 18 described HIV-1 epitopes. To assess its immunogenicity, we used four mouse strains transgenic to common HLA class II molecules (HLA-DR2, -DR4, -DQ6, -DQ8 present in 35–50% of the population), as a preclinical rodent model of the HLA class II diversity found in humans [33], [34], [35]. It has been reported that HLA class II-transgenic mice are able to develop CD4+ T cell responses to the same HLA-restricted epitopes recognized by humans carrying the same HLA class II molecule [36], [37]. Broad T cell responses were observed in all strains, covering 16 out of the 18 epitopes encoded by the DNA vaccine. We observed multiple CD4+ T cell responses restricted by several HLA-DR and –HLA-DQ molecules, as well as CD8+ T cell responses restricted to murine class I MHC. We believe this vaccine design may be potentially useful as a source of cognate T cell help to CD8+ T cell responses in novel HIV-1 vaccine candidates.

## Results

### The HIVBr18 vaccine is immunogenic and induces significant HIV-specific cytokine and proliferative T cell responses in HLA class II transgenic strains of mice

**Figure 1:**
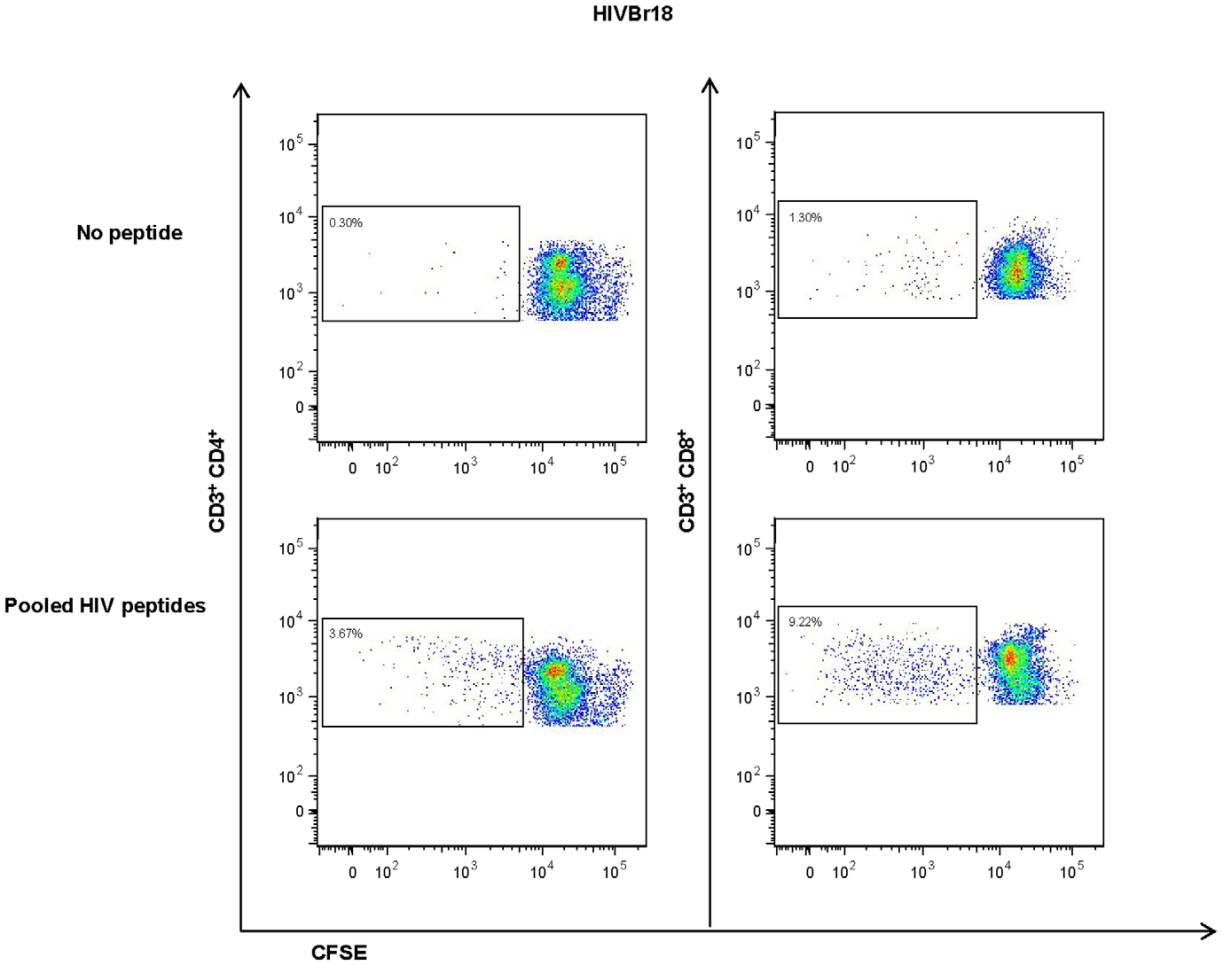
Immunization with HIVBr18 induces significant CD4+ and CD8+ T cell proliferation against pooled HIV peptides. Two weeks after the last last immunization with HIVBr18, spleen cells from a HLA-DR4 mouse were labeled with CFSE (1.25 µM) and cultured for 5 days in the presence of 5 µM of 18 pooled HIV-1 peptides. Cells were analyzed by flow cytometry and CFSE^low^ cells on gated CD3+CD4+or CD3+CD8+ was used as a readout for antigen-specific proliferation. Representative dot plots of CD4+ (left) and CD8+ (right) T cell proliferation (%CFSElow cells) of splenocytes stimulated with medium or pooled peptides from HIVBr18 immunized mice.

**Figure 2:**
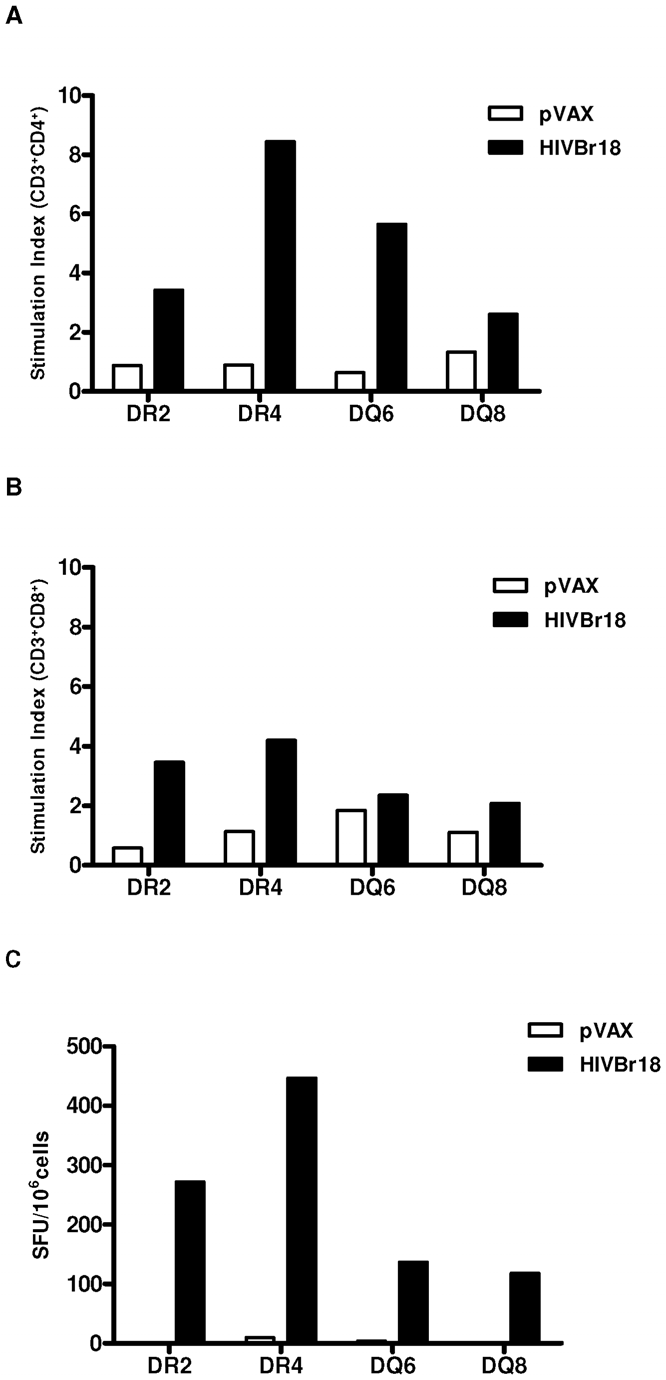
Immunization with HIVBr18 induces responses in human HLA- class II transgenic mice. Splenocytes derived from -DR2, -DR4, -DQ6 and -DQ8 transgenic mice immunized with HIVBr18 or the pVAX1 vector alone were cultured with pooled HIV-1 peptides. Proliferation of CFSE- labeled CD3^+^CD4+ (A) and CD3^+^ CD8^+^ (B) T cells from transgenic mice. The stimulation index was calculated by the fold increase between stimulated versus unstimulated cell cultures. (C) IFN-γ production by T cells from HIVBr18- (black bars) or pVAX1- (white bars) immunized mice. HLA class II-transgenic mouse strains are indicated in X axis.

To assess the magnitude and coverage of the peptide-specific response induced by the HIVBr18 vaccine in mouse strains transgenic to HLA-DR2, -DR4, -DQ6 and –DQ8, we analyzed T cell proliferation and cytokine production against pooled HIV-1 peptides. Using the CFSE (carboxyfluorescein diacetate succinimidyl ester, Molecular Probes) - based proliferation assay, we observed significant peptide-specific proliferation of both CD4+ and CD8+ splenic T cells derived from HIVBr18-immunized mice. Figure 1 depicts a representative experiment, using the DR4-transgenic strain. All four HLA class II-transgenic strains of mice presented positive proliferative responses against pooled HIV-1 peptides in splenic CD4+ T cells (Figure 2A), while strong specific CD8+ T cell responses were essentially detected only in -DR2- and DR4-Tg mice (Figure 2B). Mice from all four HLA class II-transgenic strains were able to secrete IFN-γ against pooled HIV-1 peptides as measured by ELISPOT (Figure 2C). In contrast, splenocytes from mice immunized with pVAX1 presented negligible numbers of IFN-γ secreting cells.

### Broad specific CD4+ and CD8+ T cell responses following immunization HIVBr18 in HLA class II transgenic mice

**Figure 3:**
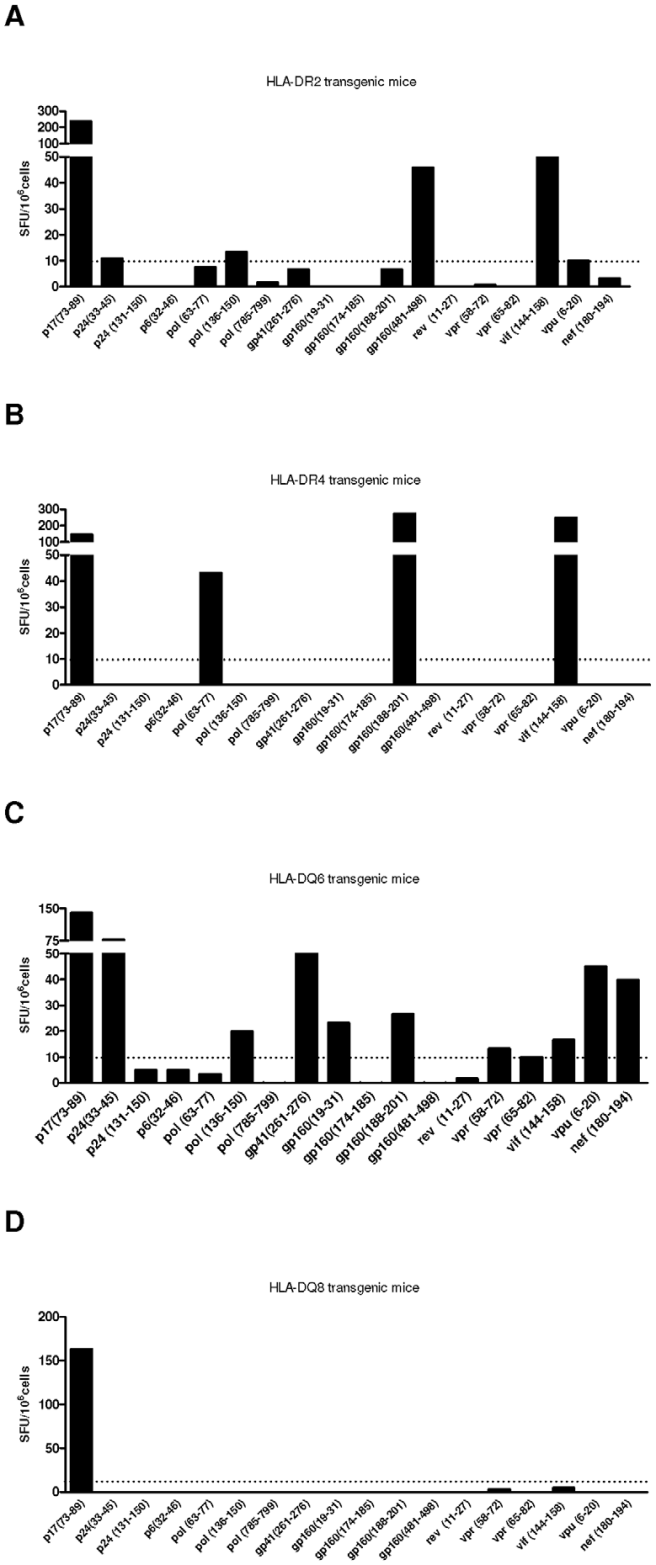
Immunization with HIVBr18 induces IFN-γ secretion against multiple epitopes in human HLA class II transgenic mice. Two weeks after the last immunization with HIVBr18 or the empty pVAX1 vector, splenocytes derived from individual HLA-DR2 (A), -DR4 (B), -DQ6 (C) and -DQ8 (D) transgenic mice (6 per group) were cultured with individual HIV-1 peptides overnight. IFN-γ production was measured by ELISPOT assay. HIV peptide-specific cellular immune responses from human HLA class II- transgenic mice that responded to the immunization are displayed. SFU, spot-forming units. Cutoff = 10 SFU/10^6^ cells and is represented by the dotted line. (SFU from pVAX1-immunized group were always below 5 SFU/10^6^ cells).

To determine whether this vaccine concept would be able to induce a broad specific immune response, splenocytes from immunized mice were incubated with each of the 18 individual HIV-1 peptides encoded by the DNA vaccine (Table S1). Immunization with HIVBr18 elicited significant numbers of IFN-γ secreting cells directed to different vaccine-encoded epitopes in HLA-DR2, -DR4, -DQ6 and -DQ8 transgenic mice, which recognized 5, 4, 10 and 1 peptides, respectively (Figures 3 A, B, C and D, respectively). Of note, the epitope p17 (73–89) induced IFN-γ secretion by spleen cells from all transgenic strains. We also evaluated the proliferative T cell responses of immunized HLA class II transgenic mice against the 18 individual peptides. Splenocytes from immunized HLA-DR2, -DR4, -DQ6 and -DQ8 transgenic mice also displayed CD4+ T cell responses to different vaccine-encoded epitopes (1, 6, 11 and 0 peptides were recognized, respectively) (Table 1). Several such responses were shared between different HLA class II-transgenic mouse strains. CD8+ T cell responses, on the other hand, were less frequent among immunized HLA-DR2, -DR4, -DQ6 and DQ8 transgenic mice (4, 2, 0 and 1 peptides, respectively) (Table 2). Taken together, HLA class II transgenic mice displayed CD4+ and CD8+ T cell responses to 11 and 6 epitopes, respectively.

**Table 1:** Immunization with HIVBr18 induces CD4+ T cell proliferation in human HLA- class II transgenic mice.

Stimulus	DR2	DR4	DQ6	DQ8
**p17(73–89)**		0.86	1.72	
**p24(33–45)**				
**p24 (131–150)**				
**p6(32–46)**			1.57	
**pol (63–77)**		0.40	1.64	
**pol (136–150)**		0.13	3.27	
**pol (785–799)**			2.54	
**gp41(261–276)**				
**gp160(19–31)**		0.35	1.42	
**gp160(174–185)**			1.35	
**gp160(188–201)**		0.31	4.33	
**gp160(481–498)**				
**rev (11–27)**			1.42	
**vpr (58–72)**				
**vpr (65–82)**				
**vif (144–158)**			3.24	
**vpu (6–20)**				
**nef (180–194)**	14.17	2.00	1.46	
cutoff	6.89	0.10	1.21	2.40
**recognized peptides**	**1**	**6**	**11**	**0**

Quantitative analysis of proliferating CD4^+^ T cells (gated on CD3^+^CD4^+^ CFSE^low^ cells) from HIVBr18 immunized transgenic mice. Proliferative responses measured 15 days after immunization. Only proliferative (%CFSE^low^) responses above cutoff are shown. Cutoff values for each transgenic strain are shown in the table. % CFSE^low^ cell values were calculated after subtraction of % CFSE^low^ cells in unstimulated cultures. Since we observed different background values in the CFSE assay among each mouse strain, individual cutoffs were established to distinguish the random noise from the true proliferation values. The nonspecific proliferative response was found to be from 0.10% to 6.89%.

**Table 2:** Immunization with HIVBr18 induces CD8+ T cell proliferation in human HLA- class II transgenic mice.

Stimulus	DR2	DR4	DQ6	DQ8
**p17(73–89)**				
**p24(33–45)**				0.87
**p24 (131–150)**				
**p6(32–46)**				
**pol (63–77)**				
**pol (136–150)**	7.94	6.94		
**pol (785–799)**	8.82			
**gp41(261–276)**		2.4		
**gp160(19–31)**	8.33			
**gp160(174–185)**	12.42			
**gp160(188–201)**				
**gp160(481–498)**				
**rev (11–27)**				
**vpr (58–72)**				
**vpr (65–82)**				
**vif (144–158)**				
**vpu (6–20)**				
**nef (180–194)**				
cutoff	7.29	0.01	12.60	0.59
**recognized peptides**	**4**	**2**	**0**	**1**

Quantitative analysis of proliferating CD8^+^ T cells (gated on CD3^+^CD8^+^ CFSE^low^ cells) from HIVBr18 immunized transgenic mice. Proliferative responses measured 15 days after immunization. Only proliferative (%CFSE^low^) responses above cutoff are shown. Cutoff values for each transgenic strain are shown in the table. For cutoff value calculation, see Table 1. The nonspecific proliferative response was found to be from 0.01% to 12.60%.

**Figure 4:**
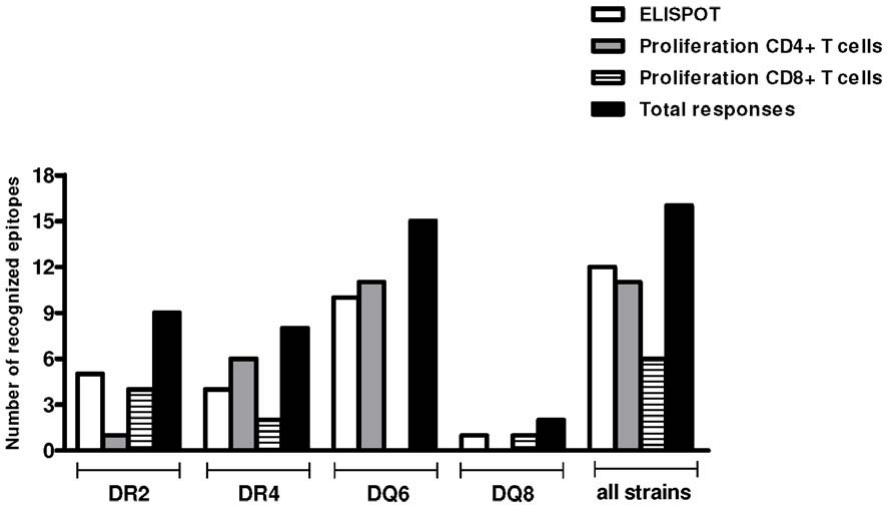
Number of recognized epitopes by spleen cells from HIVBr18 immunized HLA-DR2, -DR4, -DQ6 and -DQ8 transgenic mice. Overall peptide-specific responses observed with IFN-γ ELISPOT, CD4+ and CD8+ T cell proliferation assays in each HLA-transgenic mouse strain are depicted.

In summary, immunization with HIVBr18 was able to induce multiple T cell responses in all 4 HLA class II-transgenic mouse strains tested, against 16 out of the 18 epitopes encoded by the vaccine (Figure 4). Significantly, 11 peptides were recognized by proliferating CD4+ T cells, some of them being recognized by several HLA class II transgenic strains of mice.

## Discussion

In this report, we have developed a DNA vaccine encoding 18 conserved, multiple HLA-DR-binding HIV-1 CD4+ T cell epitopes frequently recognized by HIV-1-infected patients. The HIVBr18 vaccine was immunogenic in four mouse strains transgenic to the common HLA class II molecules HLA-DR2, -DR4, -DQ6 and -DQ8. Moreover, vaccination with HIVBr18 induced broad MHC class II-restricted T cell responses in the HLA class II-transgenic strains of mice, and 16 out of the 18 encoded epitopes could be recognized. Indeed, recent clinical trials of T cell-based HIV vaccines [9] strongly suggested that induction of broad immune responses towards conserved epitopes, in the majority of the genetically heterogeneous vaccinees, may be an essential pre-requisite for novel vaccine candidates [38], [39]. Vaccination of non-human primates with an Adenovirus 5 vaccine encoding 8 SIV proteins and devoid of Env caused a significant reduction in viral load after heterologous challenge [5], and induced broad CD4+ and CD8+ T cell responses. The breadth of pre-challenge SIV-specific T-cell responses correlated with lower viral loads and higher CD4+ lymphocyte counts [40].

An effective T cell vaccine should also have significant population coverage, given the extensive HLA polymorphism observed in human populations. In an epitope-based vaccine, one way to ensure maximum coverage is to include multiple epitopes where each one could bind to multiple HLA molecules. The TEPITOPE prediction algorithm has been successfully applied to the identification of multiple HLA-DR-binding, “promiscuous” T cell epitopes in the context of several human infectious, allergic and autoimmune diseases [41], [42], [43], [44]. A significant correlation was observed between the TEPITOPE-predicted promiscuity (ie the number of HLA-DR molecules predicted to bind to a certain peptide) and the number of HLA-DR molecules that could actually bind the peptide in biochemical assays [45]. In the conserved HIV-1 peptide set identified by our group and encoded by the HIVBr18 vaccine, each peptide could bind on average to 50% of the 9 common HLA-DR specificities tested. Conversely, most HLA-DR molecules bound to at least 10 of the peptides, indicating that an individual bearing at least one such HLA-DR molecule could develop broad CD4+ T cell responses against the HIV-1 peptide set [32]. Concerning the 6 peptides that elicited IFN- γ secretion or CD4+ T cell proliferative responses in HLA-DR2 (DRB1*1501) transgenic mice, we observed that all of them were also recognized by one or more of the 5 HLA-matched HIV-1-infected individuals tested; all 6 peptides presented binding capacity to the HLA DRB1*1501 molecule (data not shown [32]) (Table S2). Regarding the 7 peptides that elicited IFN-γ secretion or CD4+ T cell proliferative responses in HLA-DR4 transgenic mice, we observed that 3 were also recognized by the single HLA-DR4 HIV-1-infected individual tested [32] (Table S3).

The promiscuity of HLA class II binding of TEPITOPE-selected peptides may extend beyond the limited number of molecules in the prediction matrix. This is suggested by the cross-species recognition of TEPITOPE-selected peptides [45], indicating that the algorithm may select for peptides that share MHC class II binding motifs similar to many other human and non-human MHC class II molecules [31]. This may explain why so many HIV-1 infected patients and, in the present case, mice transgenic to HLA-DQ6 and -DQ8, HLA class II alleles whose binding is not predicted by the TEPITOPE algorithm, also displayed broad T cell responses to the selected HIV-1 peptide set. In addition, all TEPITOPE-selected HIV-1 peptides were predicted to bind to H-2^d^ class II molecules, and immunization of BALB/c (H-2^d^) mice with HIVBr18 also induced broad specific T cell responses to predicted peptides (unpublished observations).

The fact that each HLA class II transgenic strain recognized a different set of CD4 epitopes after immunization with HIVBr18 is in line with MHC class II-restricted recognition. Further in support of this, the fact that all peptides recognized by HLA-DR2(DRB1*1501)-transgenic mice were also recognized by at least one of the 5 tested HLA-DR15 HIV-1 infected patients [32] indicates that findings in HLA class II-transgenic mice accurately translates human responses (Table S2). The recognition of additional epitopes by some patients could possibly be explained by the expression of other HLA-DR, -DQ and -DP molecules in these individuals, contrasting to transgenic mice that carry only one HLA class II allele. In the case of HLA-DR4-transgenic mice, the fact that only 3 out of 7 recognized epitopes matched human responses is probably due to the fact that only a single HLA-DR4 HIV-1 infected patient was tested [32] (Table S3). Our finding that HLA class II transgenic strains could recognize up to 11 CD4+ T cell epitopes further indicates the ability of HIVBr18 to induce broad responses in the context of multiple HLA class II molecules. Considering that each HLA class II-transgenic strain only expresses one, rather than the 3–8 distinct class II specificities found in different HLA-DR,-DQ, and -DP haplotypes carried by humans; and the inefficient binding of HLA class II molecules with the murine CD4 molecule [46], our results indicate that the breath and magnitude of the CD4+ T cell response to the HIVBr18 immunogen may be even higher in humans. We observed that approximately 80% of mice from HLA-DR2, -DR4 and -DQ6 transgenic strains responded to the vaccination regimen, while only 30% of vaccinated HLA-DQ8 transgenic mice presented any response (data not shown). This may be due to qualitatively different levels of HLA class II expression. Regarding CD8+ T cell recognition of murine MHC class I-restricted peptides, it should be noticed that each HLA class II transgenic strain was backcrossed to mouse strains with distinct H-2 class I backgrounds [47], [48], [49].Our data confirmed the ability of the HIVBr18 immunization strategy in inducing broad HIV-1-specific proliferative and cytokine T cell responses in all HLA-DR and -DQ transgenic strains of mice tested. We observed T cell recognition of 16 out of the 18 epitopes encoded by the vaccine, thus showing that nearly all epitopes in HIVBr18 were adequately processed and presented. This implies that, on average, each strain of mice recognized epitopes corresponding to ca. 50% of the length of the insert encoded by HIVBr18. This is higher than the epitope coverage from conventional whole gene/protein HIV-1 vaccines. Although we used a CD4+ epitope-based DNA vaccine, we could also detect CD8+ T cell responses. This is not unexpected, since 78% of HIV-1 infected patients displayed CD8+ T cell responses to the pooled HIV-1 peptides, usually coexisting with CD4+ T cell responses [32]. Furthermore, 9 out of the 18 peptides contain known human or murine MHC class I-restricted CD8 epitopes [50]. To our knowledge, this is the first report of using multiple HLA class II transgenic strains of mice as a model to probe immunogenicity and HLA class II-restricted T cell responses elicited by a vaccine against an infectious disease. Data suggest that immunization with HIVBr18 might provide significant coverage of the genetically heterogeneous human population.

The significant HIV-1- specific proliferative CD4+ T cell responses in immunized mice was indicative of the magnitude of the T helper activity elicited by HIVBr18. In spite of the abundant evidence that cytotoxic CD8+ T lymphocytes (CTL) are the primary anti-HIV-1 effectors [16], a significant amount of information supports a protective role of specific CD4+ responses in HIV-1/SIV and other viral infections. Early HIV-1 specific CD4^+^ T cell responses were associated with slower progression in HIV-1 infection [19], [20]. Elite controller SIV-infected macaques mount broad CD4+-specific T cell responses, and certain macaque class II alleles are associated with significantly decreased viral loads [51]. Vaccination with the attenuated virus SIVmac239ΔNef [52] or an 8-valent SIV Adenovirus 5 vaccine [5] both induce broad, high frequency CD4+T cell responses and protect against pathogenic SIV challenge. Significantly, CD4+ T cell depletion in Rhesus macaques reduced the vaccine-induced protective effect against SIV challenge [53]. Some vaccine approaches have been especially designed to induce HIV-specific CD4+ T cells. An anti-DEC205-HIV-1 gag fusion mAb induced a CD4+ T cell response which conferred protection against challenge with recombinant vaccinia-HIV-1 gag [54]. It follows that novel immunization strategies should also aim to elicit strong CD4+ as well as CD8+ T cell responses, in order to confer long-term protective immunity [55].

We hereby demonstrate that immunization with HIVBr18, a DNA plasmid encoding a string of conserved multiple HLA class II-binding HIV-1 CD4+ T cell epitopes, can induce IFN-γ secretion, CD4+ and even some CD8+ T cell proliferation against multiple epitopes. Moreover, this T cell response was multiallelic, being able to elicit responses restricted to several distinct HLA class II molecules. Previous data from our group has shown that common HLA-DR molecules bind to multiple peptides encoded in the vaccine [32]. This indicates that T cells from individuals bearing such HLA molecules could potentially develop broad immune responses to vaccination with the conserved epitopes of HIVBr18. Thus, this vaccine concept may cope with HIV genetic variability by increasing breadth, as well as providing increased population coverage. We believe this insert design may be useful as a source of cognate T cell help in novel HIV-1 vaccine candidates.

## Materials and Methods

### Construction of DNA plasmid encoding multiple HIV-1 epitopes

We designed a multiepitopic construct containing the nucleotide sequence encoding the 18 HIV-1 epitopes described by Fonseca et al. (2006): [32] p17(73–89), p24 (33–45), p24 (131–150), p6 (32–46), protease (7–21), protease (80–94), integrase (70–84), gp41(261–276), gp160 (19–31), gp160 (174–185), gp160 (188–201), gp160 (481–498), rev (11–27), vpr (58–72), vpr (65–82), vif (144–158), vpu (6–20), nef (180–194). Epitope sequences, assembled *in tandem* in the above mentioned order, had GPGPG spacers at C and N termini, to avoid the creation of junctional epitopes and interference with processing and presentation [56]. The sequences of such epitopes are available in Supplementary Table S1. The nucleotide sequence was codon-optimized and a Kozak sequence was included at the 5′ end to improve mammalian expression. The synthetic gene was built (EZBiolab, USA, http://www.ezbiolab.com) and subcloned using *HindIII* and *XhoI* sites of the expression vector pVAX-1 (Invitrogen) for production of the recombinant DNA plasmid HIVBr18. The presence and correct orientation of the gene encoding the selected epitopes was confirmed by direct sequencing using the T7 oligonucleotide. Large-scale preparations of plasmid DNA's HIVBr18 and the empty vector pVAX1 were prepared with the Endofree® Giga Plasmid Purification Kit from Qiagen according to manufacturer's instructions. The yield and quality of purified DNA was determined by spectrophotometry at 260 nm and confirmed by agarose gel electrophoresis with ethidium bromide staining.

### Mice and Immunizations

Six to eight week-old female HLA-class II transgenic mice. DRB1* 1502 (DR2), DRB1*0401 (DR4), DQB1* 0601(DQ6) and DQB1*0302 (DQ8) were used in this study [47], [48], [49]. All transgenic mice were kindly provided by Dr. Chella S. David (Department of Immunology, Mayo Clinic, Rochester). Mice used for transgene expression were made genetically deficient for the endogenous class II genes (I-A^0^, E^0^) by homologous recombination. The expression of human HLA class II molecules on antigen-presenting cells in the thymus and periphery of these transgenic mice has a similar distribution to that of endogenous mouse MHC class II molecules [46]. Mice were kept and manipulated in SPF conditions in the animal care facilities of the Institute of Tropical Medicine, University of São Paulo (IMT/FMUSP). Experiments were performed in accordance to the guidelines of the Ethical committee of University of São Paulo. Six mice per group were injected with 10 mM cardiotoxin (Sigma) five days before vaccination. At weeks 0, 2 and 4, DNA plasmid HIVBr18 or empty vector pVAX1 was administered intramuscularly. Each quadriceps was injected with 50 µl of DNA at a concentration of 1 µg/µl in saline such that each animal received a total of 100 µg of plasmid DNA. Two weeks after the last DNA injection, mice were euthanized with CO_2_.

### Peptides

The eighteen multiple HLA-DR binding, frequently recognized peptides, derived from the conserved regions of HIV-1 B-subtype consensus and selected from the whole proteome [32] (sequences in Supplementary Table S1) were synthesized in house by solid phase technology. The 9-fluorenylmethoxycarbonyl (Fmoc) strategy, with the C- terminal carboxyl group in amide form, was used for synthesis [57]. Peptide purity and quality were assessed by reverse-phase high performance liquid chromatography and mass spectrometry and was routinely above 90%.

### Spleen cell isolation for immune assays

Two weeks after the last immunization, mice were euthanized and spleens were removed aseptically. After obtaining single cell suspensions, cells were washed in 10 ml of RPMI 1640. Cells were then ressuspended in R-10 (RPMI supplemented with 10% of fetal bovine serum (GIBCO), 2 mM L-glutamine (Sigma), 10 mM Hepes (Sigma), 1 mM sodium piruvate, 1% vol/vol non-essential aminoacid solution, 40 µg/ml of Gentamicin, 20 µg/ml of Peflacin and 5×10^−5^ M 2- mercaptoetanol (SIGMA). The viability of the cells was evaluated using 0.2% Trypan Blue exclusion dye to discriminate between live and dead cells. Cell concentration was estimated with the aid of a Neubauer chamber and adjusted in cell culture medium.

### IFNγ ELISPOT assays

Splenocytes from HIVBr18 or pVAX1 immunized mice were assayed for their ability to secrete IFN- γ after *in vitro* stimulation with 5 µM of individual or pooled HIV-1 peptides using an ELISPOT assay. The ELISPOT assay was performed using Becton Dickinson murine IFN-γ ELISPOT kit according to manufacturer's instructions. Spots were counted using an automated stereomicroscope (KS ELISPOT, Zeiss, Oberkochem, Germany). The number of antigen specific T cells, expressed as IFN-γ spot-forming units (SFU)/10^6^ splenocytes was calculated after subtracting negative control values (wells with cells in the absence of peptide). The positivity cutoff was calculated as the mean ±3 SD of splenocytes from pVAX1 immunized mice, stimulated with all peptides. The cutoff for IFN-γ was 10 SFU/10^6^ splenocytes.

### CFSE-based proliferation assay

Splenocytes from HIVBr18 or pVAX1-immunized mice were assayed for their ability to proliferate *in vitro* after stimulation with HIV-1 peptides using the CFSE dilution based proliferation assay [58]. Freshly isolated splenocytes from immunized mice were resuspended (50×10^6^/ml) in PBS, labeled with 1.25 µM of CFSE at 37°C for 10 minutes. The reaction was quenched with RPMI 1640 supplemented with 10% FBS and cells were washed before resuspending in RPMI 1640 at a density of 1.5×10^6^/ml. Cells were cultured in 96-well-round-bottomed plates (3×10^5^/well in triplicate) for 5 days at 37°C and 5%CO_2_ with medium alone or 5 µM of HIV peptides. Positive controls were stimulated with 2.5 µg/mL of Concanavalin A (Sigma). Cells were then harvested, washed with 100 µL of FACS buffer (PBS with 0.5% BSA and 2 mM EDTA) and stained with anti-mouse CD3 phycoerythrin (PE), anti-mouse CD4 peridinin chlorophyll protein (PerCP) and anti-mouse CD8 allophycocyanin (APC) (BD Pharmingen, San Jose, CA) for 45 minutes at 4°C. Cells were then washed twice with FACS buffer, fixed with 4% paraformaldehyde, and resuspended in FACS buffer. Samples were acquired on a FACSCanto flow cytometer (BD Biosciences) and then analyzed using FlowJo software (version 8.7.1, Tree Star, San Carlo, CA). Fifty thousand events (proliferation evaluation) were acquired in a live lymphocyte gate. The percent of proliferating CD4 + and CD8 +T cells, i.e., CFSE-low cells, was determined in the CD3 + cell population. The criteria for scoring as positive the proliferating cell cultures included CFSE-low cells > cut off. The cutoff of unspecific proliferative response was determined based on the median percentage of proliferating cells (% of CD3+CD4+ or CD3+CD8+ CFSE low cells) on splenocytes from pVAX-immunized groups after stimulating with individual peptides +3 standard deviations; or stimulation index >2: the stimulation index was calculated by the following equation: %CFSE^low^ cells after stimulus/%CFSE^low^ unstimulated cell cultures.

### Data Analysis

Statistical significance (p-values) was calculated by using a Student's T test or One-way ANOVA and Tukey's honestly significantly different (HSD). Statistical analysis was performed with GraphPad Prism version 4.0 software.

## Supporting Information

Table S1 Peptide sequences derived from conserved regions of B-subtype HIV-1 consensus selected for multiple HLA-DR binding by the TEPITOPE algorithm and recognition by PBMC from HIV-1-infected patients.
(0.04 MB DOC)

Table S2 Epitope recognition by HLA-DR2 transgenic mice and HIV-1-infected patients bearing the same haplotype.
(0.06 MB DOC)

Table S3 Epitope recognition by HLA-DR4 transgenic mice and HIV-1-infected patient bearing the same haplotype.
(0.04 MB DOC)
