# Supplementary material for: A Vaccine Encoding Conserved Promiscuous HIV CD4 Epitopes Induces Broad T Cell Responses in Mice Transgenic to Multiple Common HLA Class II Molecules
Source: PLoS One. 2010 Jun 11;5(6):e11072. doi: 10.1371/journal.pone.0011072 (PMC2884037; doi:10.1371/journal.pone.0011072)
Supplement: Table S1 — Peptide sequences derived from conserved regions of B-subtype HIV-1 consensus selected for multiple HLA-DR binding by the TEPITOPE algorithm and recognition by PBMC from HIV-1-infected patients. (0.04 MB DOC) [file pone.0011072.s001.doc]

**Table S1-** Peptide sequences derived from conserved regions of B-subtype HIV-1 consensus selected for multiple HLA-DR binding by the TEPITOPE algorithm and recognition by PBMC from HIV-1-infected patients.

| **Peptides** | **Sequence** | **% Recognition by PBMC from HIV-1 infected patients using IFN-γ ELISPOT assay** |
| --- | --- | --- |
| **p17(73-89)** | EELRSLYNTVATLYCVH | 31.3 |
| **p24(33-45)** | SPEVIPMFSALSE | 25.0 |
| **p24(131-150)** | KRWIILGLNKIVRMYSPTSI | 40.6 |
| **p6(32-46)** | DKELYPLASLRSLFG | 25.0 |
| **pol(63-77)** | QRPLVTIKIGGQLKE | 43.8 |
| **pol(136-150)** | TPVNIIGRNLLTQIG | 28.1 |
| **pol(785-799)** | GKIILVAVHVASGYI | 25.0 |
| **gp41(261-276)** | RDLLLIVTRIVELLGR | 28.1 |
| **gp160(19-31)** | TMLLGMLMICSAA | 31.3 |
| **gp160(174-185)** | ALFYKLDVVPID | 28.1 |
| **gp160(188-201)** | NTSYRLISCNTSVI | 21.9 |
| **gp160(481-498)** | SELYLYKVVKIEPLGVAP | 21.9 |
| **rev(11-27)** | ELLKTVRLIKFLYQSNP | 34.4 |
| **vpr(58-72)** | EAIIRILQQLLFIHF | 21.9 |
| **vpr(65-82)** | QQLLFIHFRIGCRHSRIG | 28.1 |
| **vif(144-158)** | SLQYLALVALVAPKK | 31.3 |
| **vpu(6-20)** | VLAIVALVVATIIAI | 28.1 |
| **nef(180-194)** | VLEWRFDSRLAFHHV | 28.1 |

^a^ pol (63-77) corresponds to protease (7-21)

^b^ pol (136-150) corresponds to protease (80-94)

^c^ pol (785-799) corresponds to integrase (70-84)

The use of these peptides in a vaccine formulation has been patented (international application number PCT/BR2006/000175)
