# Supplementary material for: A Vaccine Encoding Conserved Promiscuous HIV CD4 Epitopes Induces Broad T Cell Responses in Mice Transgenic to Multiple Common HLA Class II Molecules
Source: PLoS One. 2010 Jun 11;5(6):e11072. doi: 10.1371/journal.pone.0011072 (PMC2884037; doi:10.1371/journal.pone.0011072)
Supplement: Table S2 — Epitope recognition by HLA-DR2 transgenic mice and HIV-1-infected patients bearing the same haplotype. (0.06 MB DOC) [file pone.0011072.s002.doc]

**Table S2 - Epitope recognition by HLA-DR2 transgenic mice and HIV-1-infected patients bearing the same haplotype.**

|  | **-DR2 transgenic mice (DRB1* 1501)** | | **HLA-DR15 HIV-1 infected patient (IFN-γ ELISPOT; SFU/10^6^ cells))*** | | | | |
| --- | --- | --- | --- | --- | --- | --- | --- |
|  |
| **epitopes** | **IFNγ ELISPOT (SFU/10^6^ cells)** | **CD4 T cell proliferation** | **patient 1** | **patient 2** | **patient 3** | **patient 4** | **patient 5** |
| **p17(73-89)** | **+** |  |  | **+** | **+** |  |  |
| **p24(33-45)** | **+** |  | **+** | **+** |  |  | **+** |
| **p24(131-150)** |  |  | **+** | **+** | **+** |  |  |
| **p6(32-46)** |  |  |  | **+** |  |  |  |
| **pol(63-77)** |  |  |  | **+** |  |  | **+** |
| **pol(136-150)** | **+** |  |  | **+** |  |  |  |
| **pol(785-799)** |  |  |  | **+** | **+** |  |  |
| **gp41(261-276)** |  |  |  | **+** |  |  |  |
| **gp160(19-31)** |  |  | **+** | **+** |  |  |  |
| **gp160(174-185)** |  |  |  | **+** |  |  | **+** |
| **gp160(188-201)** |  |  |  | **+** |  |  | **+** |
| **gp160(481-498)** | **+** |  |  | **+** |  |  | **+** |
| **rev(11-27)** |  |  |  | **+** |  |  | **+** |
| **vpr(58-72)** |  |  |  | **+** |  |  |  |
| **vpr(65-82)** |  |  | **+** | **+** |  |  | **+** |
| **vif(144-158)** | **+** |  |  | **+** |  |  | **+** |
| **vpu(6-20)** |  |  |  | **+** |  | **+** | **+** |
| **nef(180-194)** |  | **+** | **+** | **+** |  |  | **+** |
| **recognized peptides** | **5** | **1** | **5** | **18** | **3** | **1** | **10** |

* patient data derived from ref. [32]

+: responses above cutoff.
